# The psychological empowerment and quality of work life among Jordanian primary care nurses and midwives

**DOI:** 10.3389/fmed.2024.1476225

**Published:** 2024-11-05

**Authors:** Asem Mohammad Al-Obiedat, Rabia S. Allari, Muntaha K. Gharaibeh

**Affiliations:** ^1^Faculty of Nursing/ Al-Ahliyya Amman University, Amman, Jordan; ^2^King Hussein Medical Center in Royal Medical Services, Amman, Jordan; ^3^Jordan University of Science and Technology, Faculty of Nursing/MCH Department, Irbid, Jordan

**Keywords:** Jordan, midwives, nurses, primary care, psychological empowerment, quality of work life, UN sustainability goals

## Abstract

**Introduction:**

The healthcare industry, particularly in the context of primary care, presents various challenges to nurses and midwives, influencing their psychological empowerment (PE) and quality of work life (QWL).

**Objective:**

This study’s objective is to assess the levels of PE and QWL among Jordanian primary care nurses and midwives and explore the relationship between PE and QWL.

**Methods:**

Utilizing a descriptive correlational design, the study included 273 Jordanian primary care nurses and midwives through convenience sampling. The Psychological Empowerment Instrument and Brook’s Quality of Nursing Work Life Survey were used to measure PE and QWL. Data was analyzed using descriptive statistics to describe the study participant’s characteristics and inferential analysis such as Pearson correlation, and multiple regression to examine relationships and identify predictors of study variables.

**Results:**

The study found that the QWL scale had high reliability (Cronbach’s alpha = 0.954), and similarly, the PE scale demonstrated strong reliability (Cronbach’s alpha = 0.948). Moving on to the core findings, significant positive correlations were identified between PE and QWL, with a correlation coefficient of *r* = 0.568 (*p* < 0.01), indicating that higher levels of psychological empowerment were associated with better quality of work life. Furthermore, the regression analysis revealed that PE accounted for approximately 32.3% of the variability in QWL scores. Interestingly, participants holding diplomas reported the highest QWL scores (mean = 136.14), while those contemplating leaving the nursing profession displayed significantly lower QWL scores (mean = 114.14). No significant correlations were found between PE and sociodemographic variables such as age, income, and years of experience.

**Conclusion:**

This study reveals a crucial need to enhance PE and QWL. Key findings show moderate PE and QWL levels, with variations based on educational background and workplace. The positive correlation between PE and QWL identifies the benefits of fostering empowerment through professional development, job autonomy, and decision-making. These insights are vital for improving nursing practice and policy and enhancing nurse satisfaction and patient care.

## 1 Introduction

Quality of Work Life (QWL) is defined as the degree to which registered nurses can satisfy important personal needs through their experiences in their work organization while achieving the organization’s goals (1). Quality of work life is a multifaceted variable that depicts a worker’s views about several elements of his or her job (2). Employee satisfaction, conditions of employment, appropriate and fair compensation, job advancements, work versatility, participation in decision-making tasks, occupational health and safety, workplace stress, organizational stability in employment and personal connections, and work-life continuity are examples of factors influencing workplace well-being (3, 4).

The QWL in nursing is characterized by the degree to which nursing staff may address essential individual requirements via their career experience while still achieving the organization’s mission (2). As a result of a high level of QWL, employees will feel satisfaction (5). Also, it gives people areas where they may feel relaxed, respected, and at ease (2). It has been shown that QWL impacts employee productivity in several contexts, particularly in healthcare institutions (6, 94); high QWL is necessary to attract new personnel and retain current ones (2). Therefore, greater organizational dedication and job satisfaction, effective healthcare quality, better individual and organizational productivity, lower fatigue, and individual and institutional attrition are among the potential advantages of high-level QWL (7, 92, 102).

Nurses’ QWL may fluctuate from poor to acceptable between countries (8). Akter et al. (9) reported that nurses in Bangladesh evaluated the QWL as average while 52.4% of primary health care nurses in Saudi Arabia were dissatisfied with their QWL (10), between 70.8 and 81.2% of Iranian nurses, had poor QWL (11) which was almost similar to that of Ethiopian nurses who reported that 67.2% of nurses were unsatisfied with their QWL (12). Furthermore, the setting seemed to influence the QWL; Iran and Taiwan nurses serving in outpatient units had higher QWL than staff nurses in other units (2). These differences might be attributed to the fact that inpatient nurses often work in shifts involved with direct patient care with high time constraints, job pressure, and environmental concerns, all of which lead to lower QWL (12).

Studies on QWL have identified numerous variables that affect nurses’ QWL (2, 13). One of these issues was the mismatch between work and personal life (13, 93). The top reasons for poor QWL were stressful work hours, suboptimal staffing, a loss of decision-making, handling activities irrelevant to nursing, a shortage of professional growth opportunities, an unpleasant workplace environment, and inadequate remuneration (2). Apart from these issues, management practices, interactions with coworkers, opportunities for professional growth, and work conditions all impact nurses’ QWL in the workplace (14, 100).

Another study by Yan et al. (15) highlighted that due to irregular working schedules and psychological stress, the study’s participants had to encounter work–family conflicts, which negatively influenced job performance. Prior studies in Saudi Arabia found that long working hours, incapability to manage work and family requirements, insufficient break duration, poor salaries, delay in career progression, and insufficient hospital-promoted coaching were major significant determinants for nurses’ discontentment with QWL (16). In Jordan, the study of Salahat and Al-Hamdan (17) found that most participating Jordanian nurses in the study were moderately satisfied with their QWL, and QWL was correlated positively with job satisfaction and negatively with intent to leave their job (17).

It is central to ensure the well-being of primary healthcare providers, including public health administrators, physicians, dentists, registered nurses, and midwives. These professionals should receive adequate training and be provided with the necessary biopsychosocial arrangements, as well as psychological empowerment, to effectively carry out their duties (18). Places of employment must be appealing and compassionate, and healthcare workers must be well rewarded (18, 109). Yet nurses working in primary health care are facing several stressful and unsatisfactory working circumstances (19). Nurses, for example, frequently need to pay closer attention to their health issues (2). Primary healthcare institutions are high-stress situations for both healthcare staff and patients (20). There is a constant flow of new and diverse health issues that are challenging to treat in this situation (2). These challenges require accepting responsibility for clients’ healthcare over time and caring for their diverse needs and requirements, which puts massive effort and pressure on healthcare employees, particularly nurses (20).

Psychological Empowerment (PE) refers to a psychological state in the workplace where individuals feel a sense of control, autonomy, and motivation, which enables them to engage fully in their tasks and contribute positively to their organization (21). It encompasses four key dimensions: competence, meaning, self-determination, and impact. For nurses specifically, PE takes on a distinctive meaning, embodying their ability to provide competent and compassionate patient care, find profound meaning in their role as caregivers, exercise autonomy in clinical decision-making, and understand the significant impact of their actions on patient well-being and healthcare quality. Within this context, PE becomes a driving force that motivates nurses to deliver the highest standard of care and fosters their commitment to the well-being of their patients and the healthcare system.

Studies have shown that PE has a significant role in improving nurses’ job motivation and occupational mental health (22). Higher levels of workplace empowerment were correlated with higher levels of work motivation and lower levels of occupational stress among nurses (23). PE entails emotions that indicate how much workers appreciate and contribute to the company (24, 95). Monje-Amor et al. (25) study approved that structural and psychological empowerment are critical antecedents of work engagement among employees working in Spain and the UK. Psychological empowerment may also be characterized as a process that commences with the idea that positive changes are occurring around the individual and subjectively alters employees’ perceptions of actively participating in accomplishing work-related activities with full endeavors (26). PE has four aspects: competence (the inner emotions that staff members have to do their job well); meaning (the extent to which individuals value their job”); self-determination (the extent to which staff members have control over their jobs and are allowed to select how to carry out their tasks); and impact (the extent to which staff feels they can affect their place of work) (27, 99).

Poor PE in the workplace can result in disengagement, increased stress, and reduced job satisfaction (98). A study by Jahangirian et al. (109) found that low PE was negatively correlated with the QWL among healthcare professionals, highlighting the importance of improving PE to enhance QWL and overall workplace outcomes.

Primary health care (PHC) in Jordan is provided through a network of facilities managed by the Ministry of Health (MOH). Primary health care facilities are separated into three types, Village clinics (VC), Primary Healthcare Centers (PHCs), and Comprehensive Health Centers (CHCs) (28).

The PHC provides midwifery, nursing care, and healthcare for mothers and children. In addition to general practice, a physician, and a secretary for handling administration tasks, including keeping patient-based manual medical records are also available. Primary health care’s primary responsibilities include immunizations, dentist-provided dental treatment, and a pharmacy, which supplies the most important drugs. The CHC is a state-of-the-art healthcare facility that employs a diverse team of medical professionals, including doctors, nurses (including midwives), and administrative personnel specializing in secretarial and accounting tasks. The facility has a manual record-keeping system that is organized by the family. In addition to family practice and general medicine, the CHC offers emergency treatment, which includes specialized and minor surgical services provided by MOH specialists. These experts are either permanently stationed at the center or rotate regularly. The primary areas of emphasis are immunizations, oral health treatment, medication prescriptions, laboratory services, and radiological procedures (29, 30). Jordan has 365 primary health facilities, 184 VHCs, and 122 CHCs as of 2022 (28). The Jordanian government has made universal health coverage (UHC) a strategic goal and has given it top priority in plans and initiatives. Three goals must be achieved for UHC to be realized: providing high-quality healthcare; reducing financial obstacles to healthcare access; and guaranteeing that everyone, even the most vulnerable and poor patients, has access to treatment (31). But Jordan is not at UHC yet, since several issues impact the PHC and the healthcare system. These difficulties include the changing demographics and population increase, the rising cost of treating non-communicable diseases, and the tightening of financial conditions (32).

Primary care is associated with various unpleasant and unsatisfactory work environments such as stress for workers in several professional categories, particularly nurses (19, 33). Such an environment may demonstrate that nurses who work in such extreme situations will develop low levels of QWL. The top causes of low QWL identified were stressful work schedules, staffing shortages, a manqué of autonomy in decision-making, doing jobs irrelevant to nursing, a lack of professional growth opportunities, unsuitable work conditions, inadequate wages, inadequate work hours, difficulty reconciling work with family requirements, inadequate break time, delay in promotion, and inadequate hospital-sponsored training (34, 35). After reviewing the literature, it was determined that greater attention should be devoted to the understudied issue of primary care nurses, and few studies assessed the levels of QWL among Jordanian primary care nurses. Healthcare workers, particularly community health workers, physicians, dentists, nurses, and midwives, must be cared for because individuals in these professions must be well-trained and provided with proper biopsychosocial support, and PE, to function effectively (36). Workplaces must be engaging and compassionate, and healthcare workers must be appropriately compensated (37).

Previous studies have underscored the significant role of PE in enhancing nurses’ work motivation and occupational mental health (38), with positive correlations between PE and QWL well-established in the literature. For instance, a study by Karimi et al. (97) and Jahangirian et al. (109) found a notable positive association between PE and QWL among healthcare professionals. Furthermore, it is essential to recognize that PE is not just a general concept; it has practical implications for patient care quality. Nurses who experience higher levels of PE are more likely to deliver patient-centered, high-quality care, which benefits patient outcomes (21). Nationally and regionally, many previous studies highlighted the determinants of PE among nurses, and the major factors that may influence PE were work engagement, and job security (39), feeling competence (40), management commitment (41), and Job satisfaction (42).

Enhancing the QWL is a comprehensive method to raise the QWL of workers, and every organization must acquire and retain employees (43). A strong QWL is critical for healthcare institutions to have competent, devoted, and engaged employees (110). Service provision in healthcare organizations relies on their staffing competence and skills (44). Among the several specializations in healthcare settings, nurses have a larger, more significant, and improving their QWL has emerged as a pressing issue in the healthcare industry (111). Despite the recognized importance of PE in boosting nurses’ work motivation and occupational mental health and the well-established positive correlations between PE and QWL in the existing literature, there remains a notable knowledge gap. This gap primarily pertains to the limited focus on primary care nurses specifically. While studies have explored these relationships in the broader healthcare context, there is a scarcity of research that delves into the unique challenges and dynamics faced by nurses working in primary care settings. Given primary care nurses’ distinctive demands and responsibilities, further investigation into the correlation between PE and QWL within this context is essential to provide tailored insights and strategies. Understanding this relationship among primary care nurses is crucial for addressing their specific needs and reducing intentions to leave the profession, encouraging the quality of care, and sustaining the nursing workforce in primary care settings. Closing this knowledge gap can contribute significantly to the overall well-being of primary care nurses and the healthcare system. Lastly, this study will be the first of its kind to measure Jordanian nurses’ QWL, which will aid in setting proper strategies to enhance the approach of PE. In addition, this study targets the most important priorities of sustainable development topics related to human resources development and the promotion of their integration and belonging in the workplace, thus improving productivity and effectiveness at work. In addition, the QWL is one of the most important aspects of sustainable development (45).

### 1.1 Purposes of the study

This study aims to assess the levels of PE and QWL among Jordanian primary care nurses and midwives and explore the relationship between PE and QWL.

### 1.2 Research questions

1. What is the level of PE and QWL of Jordanian primary care nurses and midwives?
2. Is there a relationship between PE, QWL and sociodemographic variables of Jordanian primary care nurses and midwives?
3. Are there differences in levels of QWL among Jordanian primary care nurses and midwives based on their selected sociodemographic variables?
4. What are the predictors of QWL of Jordanian primary care nurses?

## 2 Methodology

### 2.1 Design

The study used a descriptive cross-sectional correlational design, which is suitable for understanding how different variables relate to each other over a specific time frame. This design was chosen because it allows for a quick and straightforward way to identify connections and predict how one variable might influence another in the research (46).

### 2.2 Settings

The study took place in healthcare centers from governmental sectors in the central region of Jordan (Amman, Alzarqa, Madaba, and Albalqa). The total number of comprehensive primary health care centers in the central region, according to the MOH (28) statistics, is 41 centers. Ten healthcare centers were conveniently chosen. The selected primary healthcare centers provide comprehensive services for a wide range of the population across different age categories.

### 2.3 Population and sample

A convenient sampling method was used to select participants. The accessible population was all Jordanian primary care nurses and midwives working in selected governmental comprehensive primary care centers. The selection of this particular approach is based on its practicality, cost-effectiveness, and efficiency in targeting a certain subgroup of the population, namely primary healthcare nurses and midwives. Convenience sampling allows for the efficient collection of data from persons who are readily available and fulfill particular criteria for inclusion (47). The sample size was calculated using the G*Power 3.1.10 program. Using the regression test, the minimum required sample size was 178 (power = 0.95, *α* = 0.05, and medium effect size = 0.15 with 11 predictors). Also, 20% was added to avoid incomplete questionnaires and participants’ withdrawal. Therefore, the minimal assumed sample size required was 213. The inclusion criteria for the sample selection include only nurses and midwives who (a) are full-time employees, (b) are employed for a minimum of 6 months, and are involved in direct client care. Those on leave during the study period and nurse managers were excluded from the study. Although this study is descriptive, confounders were managed through inclusion criteria: participants must be currently employed at PHC centers at the time of the data collection to capture the insights of current practices and challenges in primary healthcare settings; with a minimum of 6 months of experience to ensures participants have substantial exposure to the primary healthcare environment, making their contributions to understanding the quality of work life and psychological empowerment more valuable. In addition, the researcher made sure that the sample had the sufficient number of nurses and midwives for measuring differences from different PHCs in the central region of Amman with diverse sociodemographic characteristics.

### 2.4 Study instruments

A self-reported paper-based questionnaire was distributed to nurses and midwives. The survey package consisted of three sections: the sociodemographic part, the PE instrument, and Brook’s Quality of Nursing Work Life Survey.

Sociodemographic Questions. It includes age, gender, marital status, monthly income, educational level, job title, years of experience, weekly working hours, overtime, an intention to leave the current workplace, and intention to leave the nursing career.

Psychological Empowerment Instrument: The original instrument was developed by Spreitzer (21) to assess nurses’ psychological empowerment. The scale is composed of 12 items equally distributed in four sub-dimensions (meaning, competence, self-determination, and impact). The mean of the four sub-dimensions was used to create an overall empowerment score. Responses to each statement are rated on a 5-point Likert scale, ranging from 1 (strongly disagree) to 5 (strongly agree). The overall total score ranges from 12 to 60, and the subscale total score ranges from 3 to 15, where a higher score means a higher level of psychological empowerment. The PE instrument reported good validity and reliability (21, 48, 49), and the internal consistency reliability of the original scale using Cronbach’s *α* was 0.80 (21, 48). The PE 5 points Likert scale (49) was originally developed in English and was translated to Arabic by Malak and Abu Safieh (50), who concluded that the tool is valid after testing for face validity and showed Cronbach’s alpha for internal consistency of 0.87, and this Arabic version was adopted in this study after guaranteeing permission of the authors.

Brook’s Quality of Nursing Work-Life Survey (BQNWLS): The scale was originally developed in the English language by Brooks (51) to measure nurses’ work-life quality. The BQNWLS is a 42-item scale that enables respondents to rate their level of agreement or disagreement on a six-point scale ranging from 1 (strongly disagree) to 6 (strongly agree). The overall score for the BQNWLS is calculated by summing the values of all 42 questions, and it ranges from 42 to 252, with a higher number reflecting superior QWL. The internal consistency coefficient of the original BQNWL scale was 0.89 (51). The BQNWL was translated for this study into Arabic (108). A pilot study was conducted on a sample of 30 primary care nurses from different centers The BQNWL scale demonstrated a high internal consistency with a Cronbach’s alpha coefficient of 0.95, indicating a reliable measurement instrument.

### 2.5 Ethical considerations

Approval of the study was obtained from the institutional review board (IRB) of where the researchers work No. (MOH\REC\2023\98). Nurses and midwives were informed that participation in this study is voluntary and no penalties for non-participation. They were also assured that they had the right not to answer the question they chose. Participants were instructed that completing and returning the questionnaire would be considered as written consent for participation. The cover letter attached to each questionnaire included an explanation about the research purpose and assured them of the confidentiality of the obtained information, which will be used by the researcher only, and no risks are associated with the completion of the survey. The permission of the authors of the translated instruments was obtained prior to the data collection process. The collected data was saved on the investigator’s password-protected personal computer. All processes in this study adhere to the institutional research committee’s ethical guidelines as well as the 1964 Helsinki Declaration and its subsequent revisions or similar ethical standards (52).

### 2.6 Data collection process

The data collection process lasted for 6 weeks from Feb to mid-April 2023. The managers of the ten selected healthcare centers were approached by the researcher who explained the study’s purpose and asked for their support in the data collection process. The researcher and the manager verified the number of nurses and midwives in each selected center. The researcher then approached nurses and midwives in their workplaces and distributed the questionnaire to those who met the criteria. The questionnaire, accompanied by an envelope and a cover letter was distributed by the primary researcher to all nurses in each center after they agreed to participate. Nurses and midwives were asked to complete the questionnaire, put it in a sealed envelope, and return it to a box that was designated for the completed one.

The investigator personally collected the sealed envelopes every 3 days until the end of the data collection time. A code, known only to the investigator, was placed on each completed envelope in the top right corner of the front sheet to note the center. To maintain strict confidentiality of responses, the codes were removed from the questionnaires when the analysis was completed. A total of 320 envelopes were distributed to the ten sites, and 290 questionnaires were returned to the boxes in the 10 centers giving a response rate of 90.6%.

### 2.7 Data analysis

The IBM SPSS version 22 was used to analyze data from the 290 participants. Data was cleaned and checked for any missing information or outliers. Descriptive statistics are used to describe and analyze a dataset’s main features and characteristics without making any generalizations or inferences to a larger population. Descriptive statistics of the participant’s demographic data were computed using central tendency and dispersion measures. Continuous variables like levels of perceived PE and QWL were measured using Mean (M) and Standard deviation (SD) for each item and total scale items, and frequency for categorical variables. Subscales’ means were calculated. Relationships between PE, participants’ sociodemographic characteristics, and QWL were tested using the Pearson correlation test (r) (46). The differences in averages of QWL were examined via t-test and one-way ANOVA. ANOVA is a versatile and powerful statistical technique and an essential tool when researching multiple groups or categories. The one-way ANOVA can help you know whether or not there are significant differences between the means of your independent variable, and the t-test is an inferential statistic used to determine if there is a significant difference between the means of two groups and how they are related (46). Also, A *post hoc* analysis was conducted to determine the group comparisons. Multiple linear regression was utilized to identify predictors of QWL. The *p*-value <0.05 was considered statistically significant.

## 3 Results

### 3.1 Demographic descriptive statistics

The mean age of the participants was 33.30 years (SD = 7.31), The participants’ monthly incomes mean is 526.28 dinars (SD = 146.11). The participants had varying levels of experience in the nursing profession, with an average of 11.75 years (SD = 7.11). Similarly, the years of employment in the current health center mean is 5.99 years (SD = 5.30) as indicated in Table 1.

**Table 1:** Demographic and work-related characteristics of participants (*N* = 273).

Variable	Minimum	Maximum	*M*	SD
Age	20.00	57.00	33.30	7.31
Monthly income in Jordanian dinars	200.00	1100.00	526.28	146.11
Years of experience	1.00	36.00	11.75	7.11
Years of employment in the current health center	1.00	29.00	5.99	5.30
Weekly working hours	36	72.00	42.01	7.35
Variable			F	%
Gender				
Male			53	19.4
Female			220	80.6
Marital status				
Single			68	24.9
Married			197	72.2
Others			8	3.0
Educational level				
Diploma			106	38.8
Bachelor			132	48.4
Postgrad			35	12.8
Job title				
Registered nurse			232	85.0
Midwife			41	15.0
Over time				
Yes			39	14.3
No			234	85.7
Intention to leave current workplace				
Yes			53	19.4
No			220	80.6
Reasons for Intention to leave current workplace				
Inconvenient working condition			140	51.3
For professional development opportunities			133	48.7
Intention to leave nursing career				
Yes			49	17.9
No			224	82.1
Reasons for intention to leave nursing career				
Work-related Challenges			182	66.7
Financial and Social Factors			91	33.3

M, mean; SD, standard deviation; F, frequency; %, percentage.

On average, the participants reported working 42.01 h per week (SD = 7.35), with weekly working hours ranging from 36 to 72.00 h. In terms of gender distribution, 53 participants (19.4%) were male, while the majority, 220 participants (80.6%), were female. 106 participants (38.8%) held a diploma, 132 participants (48.4%) had a bachelor’s degree, and 35 participants (12.8%) had a postgraduate degree. Among the participants, the majority, 232 individuals (85.0%), held the job title of registered nurse, while 41 individuals (15.0%) were midwives. A total of 39 participants (14.3%) reported working overtime, while the remaining 234 participants (85.7%) did not engage in overtime work.

When it comes to their intentions, 53 participants (19.4%) expressed an intention to leave their current workplace, while 220 participants (80.6%) reported no such intention. Among those with an intention to leave, the most cited reason was inconvenient working conditions, mentioned by 140 individuals (51.3%). In comparison, 133 individuals (48.7%) expressed that the lack of professional development opportunities was a contributing factor. Additionally, 49 participants (17.9%) indicated an intention to leave the nursing career, while the majority, 224 participants (82.1%), expressed no such intention.

### 3.2 The levels of PE and QWL of Jordanian primary care nurses and midwives

Table 2 provides descriptive statistics for the PE scale and its subscales among the same participants. The PE total scale scores ranged from 12.00 to 60.00, with a mean score of 43.01 (SD = 12.03). On the PE meaning subscale, scores ranged from 3.00 to 15.00, with a mean score of 10.80 (SD = 3.22). For the PE competence subscale, scores ranged from 3.00 to 15.00, with a mean score of 11.17 (SD = 3.20). The PE self-determination subscale had scores ranging from 3.00 to 15.00, with a mean score of 10.35 (SD = 3.37). Lastly, on the PE self-impact subscale, scores ranged from 3.00 to 15.00, with a mean score of 10.68 (SD = 3.16).

**Table 2:** Descriptive statistics for PE scales and subscales (*N* = 273).

Variable	Min	Max	Mean	Std. Deviation
PE total scale	12.00	60.00	43.01	12.03
PE: meaning subscale	3.00	15.00	10.80	3.22
PE: competence subscale	3.00	15.00	11.17	3.20
PE: self-determination subscale	3.00	15.00	10.35	3.37
PE: self-impact subscale	3.00	15.00	10.68	3.16

PE, psychological empowerment.

Table 3 presents descriptive statistics for items on the PE scale. The top-rated item, on average, was “I am confident in my ability to do my job,” with a mean score of 3.75. Following closely is the statement, “The work I do is important to me,” with a mean score of 3.6557. “I have significant autonomy in determining how I do my job” also received a high mean score of 3.52. “My impact on what happens in my department is large” and “My job activities are personally meaningful to me” received mean scores of 3.53 and 3.47, respectively. Conversely, the lowest-rated item on the scale is “I can decide on my own how to go about doing my own work,” with a mean score of 3.4103. “I have considerable opportunity for independence and freedom in how I do my job” received a slightly higher mean score of 3.42 but still falls among the lower-rated items.

**Table 3:** Psychological empowerment items (*N* = 273).

Items	Minimum	Maximum	Mean	Std. Deviation
I am confident in my ability to do my job	1.00	5.00	3.78	1.38
The work I do is important to me	1.00	5.00	3.65	1.31
I have significant autonomy in determining how I do my job	1.00	5.00	3.52	1.25
My impact on what happens in my department is large	1.00	5.00	3.53	1.23
My job activities are personally meaningful to me	1.00	5.00	3.47	1.20
I have a great deal of control over what happens in my department	1.00	5.00	3.50	1.22
I can decide on my own how to go about doing my own work	1.00	5.00	3.43	1.30
I have considerable opportunity for independence e and freedom in how I do my job	1.00	5.00	3.42	1.32
I have mastered the skills necessary for my job	1.00	5.00	3.72	1.19
The work I do is meaningful to me	1.00	5.00	3.67	1.20
I have significant influence over what happens in my department	1.00	5.00	3.64	1.21
I am self-assured about my capabilities to perform my work activities	1.00	5.00	3.65	1.20

Table 4 presents the descriptive statistics for the BQNWL scale and its subscales among the participants (*N* = 273). The BQNWL total score ranged from 45.00 to 205.00, with a mean score of 130.92 (SD = 30.19). The participants’ scores on the BQNWL Work Life subscale ranged from 7.00 to 30.00, with a mean score of 17.85 (SD = 5.56). For the QWL Work Design subscale, the scores ranged from 12.00 to 50.00, with a mean score of 33.06 (SD = 7.66). The BQNWL Work Context subscale had scores ranging from 19.00 to 95.00, with a mean score of 62.30 (SD = 15.40). Finally, on the BQNWL Work World subscale, scores ranged from 5.00 to 25.00, with a mean score of 14.86 (SD = 4.51).

**Table 4:** Descriptive statistics for BQNWL scale and subscales (*N* = 273).

Variable	Min	Max	Mean	Std. Deviation
BQNWL total score	45.00	205.00	130.92	30.19
BQNWL Work Life subscale	7.00	30.00	17.85	5.56
BQNWL work design subscale	12.00	50.00	33.06	7.66
BQNWL Work Context subscale	19.00	95.00	62.30	15.40
BQNWL Work World subscale	5.00	25.00	14.86	4.51

BQNWL, Brooks Quality of Nurse Work life.

The highest-ranked items in the BQNWL scale reveal significant insights into the aspects of the work environment that healthcare professionals value the most. Notably, respondents gave the highest mean score to the statement “I feel like there is teamwork in my work setting” (Mean = 3.43). This result underscores the importance of teamwork in healthcare, indicating that employees perceive a strong sense of collaboration and mutual support among their colleagues. Additionally, “Friendships with my co-workers are important to me” and “I feel like I belong to the ‘work family’“received identical mean scores of 3.52. Finally, “I am able to communicate with other therapists” garnered a mean score of 3.66, indicating that healthcare professionals believe in effective communication with their peers in related fields, which is essential for delivering coordinated and high-quality patient care. These findings highlight the significance of positive interpersonal relationships, teamwork, and effective communication as crucial components of a satisfying and fulfilling work environment in the healthcare sector (Table 5).

**Table 5:** Items for the BQNWL scale (*N* = 273).

	Mean	Std. Deviation
I am able to balance work with my family needs	3.22	1.25
I am able to arrange for child-care when I am at work	2.95	1.24
I have energy left after work	2.86	1.27
My organization’s policy for family-leave time is adequate	2.94	1.27
I am able to arrange for day care for my elderly parents	2.86	1.23
I am able to arrange for day care when my child is ill	2.97	1.21
I receive a sufficient amount of assistance from unlicensed support personnel	3.30	1.20
I am satisfied with my job	3.41	1.18
My workload is too heavy	3.23	1.27
I have autonomy to make patient care decisions	3.37	1.21
I perform many non-nursing tasks	3.31	1.27
I experience many interruptions in my daily work routine	3.25	1.19
I have enough time to do my job well	3.19	1.27
There are enough RNs in my work setting	3.03	1.32
I am able to provide good quality patient care	3.60	1.2
I receive quality assistance from unlicensed support personnel	3.33	1.27
I am able to communicate well with my nurse manager/supervisor	3.35	1.22
I have adequate patient care supplies and equipment	3.20	1.26
My nurse manager/supervisor provides adequate supervision	3.35	1.20
Friendships with my co-workers are important to me	3.52	1.15
My work setting provides career advancement opportunities	3.29	1.25
I feel like there is teamwork in my work setting	3.43	1.18
I feel like I belong to the “work family	3.52	1.15
I am able to communicate with other therapists (physical, respiratory, etc.)	3.66	1.06
I receive feedback on my performance from my nurse manager/supervisor	3.35	1.18
I am able to participate in decisions made by my nurse manager/supervisor	3.35	1.17
I feel respected by physicians in my work setting	3.57	1.16
The nurses’ lounge/break-area/locker room in my setting is comfortable	2.84	1.34
I have access to degree completion programs through my work setting	2.85	1.30
I receive support to attend in-services and continuing education programs	3.10	1.32
I communicate well with the physicians in my work setting	3.42	1.22
I am recognized for my accomplishments by my nurse manager/supervisor	3.19	1.19
Nursing policies and procedures facilitate my work	3.23	1.19
I feel the security department provides a secure environment	3.05	1.25
I feel safe from personal harm (physical, emotional, or verbal) at work	3.01	1.31
I feel that upper-level management has respect for nursing	2.78	1.32
I believe that, in general, society has the correct image of nurses	2.68	1.29
My salary is adequate for my job given the current job market conditions	2.54	1.32
I would be able to find the same job in another organization with about the same salary and benefits	3.05	1.23
I feel my job is secure	3.26	1.22
I believe my work impacts the lives of patients/families	3.31	1.33

On the contrary, the lowest-ranked items in the BQNWL scale shed light on areas of concern and dissatisfaction among healthcare professionals. Notably, the statement “My salary is adequate for my job given the current job market conditions” received the lowest mean score (Mean = 2.54). This result suggests that, on average, respondents do not feel that their salaries are commensurate with the demands of their positions, considering the current job market conditions. This financial concern can contribute to stress and job dissatisfaction among healthcare workers. Similarly, “I believe that, in general, society has the correct image of nurses” received a relatively low mean score of 2.68, indicating that respondents, on average, do not believe that society holds an accurate or favorable perception of the nursing profession. This perception could potentially affect morale and job satisfaction, as it reflects concerns about the recognition and appreciation of nursing in the broader community. Additionally, “I feel that upper-level management has respect for nursing” garnered a mean score of 2.78. Moreover, “The nurses’ lounge/break-area/locker room in my setting is comfortable” and “I have access to degree completion programs through my work setting” received mean scores of 2.84 and 2.85, respectively, indicating that respondents are dissatisfied with the comfort of their physical workspaces and the availability of educational opportunities through their workplaces. These factors contribute to the overall work environment and can impact on job satisfaction and well-being.

### 3.3 The relationship between PE, QWL, and sociodemographic variables of Jordanian primary care nurses and midwives

A significant positive moderate correlation was observed between PE and the BQNWL total score (*r* (271) = 0.568, *p* < 0.01) as indicated in Table 6, highlighting the robust connection between psychological empowerment and the overall quality of work life in this healthcare setting. Specifically, the BQNWL Work Design subscale exhibited the strongest positive correlation with the PE total score (*r* (271) = 0.628, *p* < 0.01), underscoring the crucial role of work task and responsibility design in influencing nurses’ psychological empowerment. Additionally, the BQNWL Work Life subscale (*r* (271) = 0.510, *p* < 0.01) emphasized the significance of work-life balance with psychological empowerment, while the BQNWL Work Context subscale (*r* (271) = 0.497, *p* < 0.01) highlighted the impact of the work environment on nurses’ empowerment.

**Table 6:** The relationship between PE and QWL among Jordanian primary care nurses and midwives (*N* = 273).

Variable		QWL total score	QWL work life subscale	QWL work design subscale	QWL work context subscale	QWL work world subscale
P.E total score	Pearson Correlation	0.568^**^	0.510^**^	0.628^**^	0.497^**^	0.361^**^
	Sig. (2-tailed)	0.000	0.000	0.000	0.000	0.000
P.E meaning subscale	Pearson Correlation	0.520^**^	0.467^**^	0.569^**^	0.465^**^	0.311^**^
	Sig. (2-tailed)	0.000	0.000	0.000	0.000	0.000
P.E competence subscale	Pearson Correlation	0.568^**^	0.497^**^	0.640^**^	0.498^**^	0.357^**^
	Sig. (2-tailed)	0.000	0.000	0.000	0.000	0.000
P.E self-determination subscale	Pearson Correlation	0.535^**^	0.516^**^	0.584^**^	0.451^**^	0.351^**^
	Sig. (2-tailed)	0.000	0.000	0.000	0.000	0.000
P E self-impact subscale	Pearson Correlation	0.487^**^	0.411^**^	0.538^**^	0.432^**^	0.322^**^
	Sig. (2-tailed)	0.000	0.000	0.000	0.000	0.000

r, Pearson correlation coefficient; P, Significance.

** Correlation is significant at the 0.01 level.

The correlation analysis between selected demographic variables with the QWL and PE total score (*N* = 273) revealed that age, monthly income in Jordanian dinars, years of experience, and years of employment in the current health center did not show significant correlations with the QWL or PE total score. The correlations were all non-significant (*p* > 0.05), suggesting that these demographic factors did not have a notable relationship with the level of PE among Jordanian primary care nurses and midwives in this study.

### 3.4 The differences in levels of QWL among Jordanian primary care nurses and midwives based on their selected sociodemographic variables

Table 7 presents the variations in QWL levels based on selected demographic characteristics of Jordanian primary care nurses and midwives (*N* = 273). For the variable “Educational level,” the mean QWL scores differed significantly among the groups (*p* = 0.02). Participants with a diploma had the highest mean QWL score (136.14), followed by those with a postgraduate degree (134.91), and those with a bachelor’s degree (125.67). No significant differences in QWL scores were found based on gender (*p* = 0.23), marital status (*p* = 0.65), specialty (*p* = 0.18), or overtime work (*p* = 0.09).

**Table 7:** Variations in QWL levels based on selected demographic characteristics of Jordanian primary care nurses and midwives (*N* = 273).

Variable	*N*	Mean	Sig
**Educational level**			0.020
Diploma	106	136.14	
Bachelor	132	125.67	
Postgraduate	35	134.91	
**Gender**			0.233
Male	53	135.56	
Female	220	129.80	
**Marital status**			0.653
Single	72	129.50	
Married	201	131.43	
**Specialty**			0.185
Nursing	232	129.82	
Midwives	41	137.17	
**Over time**			0.095
Yes	39	139.07	
No	234	129.56	
**Intention to leave the current workplace**			0.021
Yes	53	122.05	
No	220	133.05	
**Reasons for intention to leave the workplace**			0.974
Inconvenient working condition	140	130.86	
For professional development opportunities	133	130.98	
**Intention to leave the nursing profession**			0.000
Yes	49	114.14	
No	224	134.59	
**Reasons for intention to leave the nursing profession**			0.967
Work-related Challenges	182	130.97	
Financial and Social Factors	91	130.81	

However, significant differences in QWL scores were observed for the variable “Intention to leave current workplace” (*p* = 0.02). Participants intending to leave their current workplace had a lower mean QWL score (122.05) compared to those who did not express such intention (133.05). Significant differences in QWL scores were also found for the variable “Intention to leave nursing profession” (*p* = 0.000). Participants with an intention to leave the nursing profession had a significantly lower mean QWL score (114.14) compared to those who did not have such an intention (134.59). No significant differences in QWL scores were observed based on the reasons for intention to leave the workplace (*p* = 0.97) or the reasons for intention to leave the nursing profession (*p* = 0.96).

*Post hoc* test results in Table 8 showed that nurses and midwives with a diploma had significantly higher QWL scores compared to those with a bachelor’s degree (mean difference: 10.46, *p* = 0.015), and those with a postgraduate degree showed significantly higher scores than bachelor’s degree holders (mean difference: 9.24, *p* = 0.025). Regarding the intention to leave the current workplace, those without the intention had significantly higher QWL scores than those intending to leave (mean difference: 11.01, *p* = 0.021). Similarly, for the intention to leave the nursing profession, both nurses and midwives without such intention had significantly higher QWL scores than those with the intention (mean differences: 20.45 for nursing, 17.63 for midwives, *p* < 0.05 in both cases).

**Table 8:** *Post hoc* test results for QWL based on selected variables among Jordanian primary care nurses and midwives (*N* = 273).

Variable	Subgroup comparison	Mean difference	*p*-value	Significance	Significant subgroup
Educational level	Diploma vs. Bachelor	10.46	0.015	Significant	Diploma
Educational level	Bachelor vs. Postgraduate	9.24	0.025	Significant	Postgraduate
Intention to leave the current workplace	Yes vs. No	11.01	0.021	Significant	No Intention
Intention to leave the nursing profession	Yes vs. No (Nursing)	20.45	0.000	Significant	No Intention
Intention to leave the nursing profession	Yes vs. No (Midwives)	17.63	0.010	Significant	No Intention

### 3.5 Predictors of QWL among Jordanian primary care nurses and midwives

The multiple linear regression analysis was conducted to examine the predictors of QWL among Jordanian primary care nurses and midwives. The overall model summary indicates that the single predictor variable, “PE total score,” accounts for a significant proportion of the variance in QWL (R Square = 0.32). The adjusted R Square value, which takes into consideration the number of predictors in the model, is 0.32. This suggests that approximately 32.3% of the variability in QWL scores can be explained by the “PE total score.”

The standard error of the estimate is 24.8, reflecting the average difference between the observed and predicted QWL scores. The F-statistic of 129.3 is highly significant (*p* < 0.001), indicating that the model is a good fit for the data. The Durbin-Watson statistic of 1.75 falls within the acceptable range, suggesting that there is no significant autocorrelation in the residuals.

In terms of the individual predictor variable, the unstandardized coefficient for “PE total score” is 1.42 (SE = 0.12), indicating the estimated increase in QWL score associated with a one-unit increase in the “PE total score.” The standardized coefficient (Beta) for this predictor is 0.56, which is statistically significant (*t* = 11.3, *p* < 0.001). This Beta value suggests that the “PE total score” is a robust contributor to the model, explaining a substantial portion of the variance in QWL scores.

Collinearity diagnostics were conducted to assess multicollinearity among the predictor variables. The tolerance value for the single predictor variable is 1.000, while the variance inflation factor (VIF) value is also 1.000. These results indicate that there is no evidence of multicollinearity, as there was only one predictor variable in the model.

## 4 Discussion

### 4.1 Levels of PE and QWL among nurses

The current study findings demonstrate that the PE and QWL levels among Jordanian primary care nurses and midwives need improvement. Although the mean score for the PE total scale was 43.0147, which is slightly above the midpoint of the scale, the scores for the PE subscales were considered moderate. The highest score was found in the competence subscale, indicating that nurses and midwives perceived their abilities and skills to be the most empowering aspect of their work life. The low scores on the meaning and self-determination subscales suggest that the nurses feel a lack of purpose and autonomy in their work. The low scores on the meaning and self-determination subscales of the PE instrument in this study are consistent with previous research on nursing job satisfaction, which has shown that nurses often experience a lack of control and autonomy in their work (53–56). The nursing profession is known to be highly demanding and stressful, which can lead to feelings of burnout and a lack of meaning in work (57–59). The current study findings highlight the need for interventions that support the development of nurses’ sense of meaning and autonomy in their work to improve their PE.

Regarding QWL, the mean score for the total scale was 130.92, which is below the midpoint of the scale, and according to Brooks (51), it falls into the moderate category. This suggests that Jordanian primary care nurses and midwives perceive their work life to be of low quality. The highest score was found in the Work Context Dimension subscale, indicating that the work environment and resources were perceived to be the most favorable aspects of their work life. On the other hand, the lowest score was found in the Work World Dimension subscale, indicating that nurses and midwives perceived their workload and job demands to be the least favorable aspect of their work life. The possible reasons for the low QWL scores in this study could be due to the high workload and stress associated with the nursing profession, which may lead to feelings of job dissatisfaction and burnout (60–62). Furthermore, the low QWL scores may be due to the lack of resources and support for nurses in the primary care setting in Jordan (63), such as limited opportunities for professional development and advancement (64), inadequate staffing levels, and inadequate compensation (65).

The findings of this study have several novel contributions to the existing literature. First, this study contributes to the literature by providing insights into the level of empowerment among primary care nurses in Jordan. The study’s finding that the mean score for the PE total scale was slightly above the midpoint of the scale suggests that nurses and midwives in primary care settings in Jordan perceive themselves to be moderately empowered. This result is important as it highlights the need for interventions to enhance the level of empowerment among nurses and midwives in Jordan.

Second, the study’s finding that the highest score was found in the competence subscale and the lowest score was found in the self-determination subscale is novel in the context of Jordanian primary care nurses and midwives. While previous studies have examined the relationship between psychological empowerment and job satisfaction or turnover intention among nurses in Jordan (27, 42, 66, 101), few studies have focused specifically on primary care nurses and midwives. Furthermore, few studies have examined the subscales of psychological empowerment and their relationship with quality of work life among nurses and midwives in Jordan. Therefore, this study contributes to the literature by shedding light on the specific aspects of PE that are most and least empowering for Jordanian primary care nurses and midwives.

Third, the study’s finding that the QWL scores for Jordanian primary care nurses and midwives were moderate suggests that there is room for improvement in the QWL among this population. This finding is novel in the context of Jordanian primary care nurses and midwives as few studies have examined the quality of work life among this population. Therefore, this study contributes to the literature by providing insights into the specific dimensions of QWL that are most and least favorable for Jordanian primary care nurses and midwives.

In addition, the results of this study revealed that the determinants of PE among nurses in Jordan were not different from those nurses at regional or even international levels, which approve that the nursing profession must have certain environmental characteristics that support the nurses and improve the quality of work life which accordingly will support the nurse perception of psychological empowerment.

### 4.2 The relationship between PE, sociodemographic variables, and QWL among nurses

The findings of this study reveal a significant positive relationship between PE and QWL among Jordanian primary care nurses and midwives. This suggests that an increase in the level of PE corresponds to an improvement in QWL. This finding aligns with existing research in nursing and other fields, which has consistently demonstrated the positive associations of PE with various work-related outcomes, including job satisfaction (67, 68), organizational commitment (69), and work engagement (70). These positive outcomes have been linked to enhanced productivity, better patient outcomes, and lower turnover rates within the nursing profession (71).

Furthermore, it’s noteworthy that the significant positive correlation between PE and QWL is a novel finding within the context of Jordanian primary care nurses and midwives. While previous research has explored the relationship between PE and job satisfaction across various healthcare settings (42, 72–74), few studies have specifically investigated the connection between PE and QWL in this particular population. Therefore, this study contributes valuable insights to the literature on employee well-being and sheds light on the factors that influence the QWL of primary care nurses and midwives in Jordan.

Nonetheless, it is important to acknowledge that not all studies have consistently demonstrated such strong associations between PE and work-related outcomes. Some research has revealed weaker or non-significant relationships between PE and factors like job satisfaction (75) and work engagement (76). Additionally, these findings suggest that while PE plays a significant role in fostering positive work-related outcomes, it may not be the sole determinant. Other factors, such as job resources (e.g., social support, job autonomy, feedback), have been identified as important predictors of job satisfaction and work engagement (77, 78). Hence, while the current study underscores the positive correlation between PE and QWL, it is essential to recognize that multiple factors can collectively influence work-related well-being.

Furthermore, the study’s observation of a weak negative correlation between PE and weekly working hours suggests that extended working hours may negatively impact nurses’ PE. This finding is consistent with prior research indicating that long working hours can lead to fatigue, stress, and burnout, subsequently diminishing nurses’ sense of empowerment and work satisfaction (45). Additionally, extended working hours can disrupt the work-life balance, limiting opportunities for nurses to engage in activities that contribute to their well-being, such as spending time with family and friends or pursuing leisure activities (79–81).

However, it’s important to acknowledge that some studies have reported no significant relationship between working hours and job satisfaction among nurses (82). These inconsistencies may stem from variations in study design, sample characteristics, and cultural and organizational factors.

Additionally, the weak positive correlation between QWL total scores and age implies that older nurses may experience better QWL. This observation may be attributed to the fact that older nurses typically have more experience and, as a result, may enjoy higher job satisfaction and job security (83). Overall, the study’s findings underscore the potential for interventions aimed at enhancing PE to also improve the QWL of Jordanian primary care nurses and midwives. These insights are crucial for healthcare organizations when designing strategies to enhance employee well-being and overall productivity.

### 4.3 Differences in levels of QWL among nurses

The results of this research indicate that certain sociodemographic factors might influence the QWL of primary care nurses and midwives in Jordan. The results of this research indicate that certain sociodemographic factors influence the QWL of primary care nurses and midwives in Jordan. The results indicate that nurses with a diploma exhibited notably superior QWL ratings in comparison to those possessing a bachelor’s degree. This finding may be attributed to the fact that nurses with higher than diploma degrees often undergo more extensive practical training, acquire higher experience, have more administrative responsibilities and burdens according to their job description, and more workload, resulting in a heightened feeling of competence and work satisfaction (84), accordingly increasing their quality of work-life needs, if not met, it leads to dissatisfaction. Additionally, nurses with bachelor’s degrees encounter a greater number of administrative and bureaucratic duties (85), resulting in less time dedicated to patient care and a worse feeling of job satisfaction (86). The results align with other studies that indicated a positive correlation between nurses’ educational attainment and their reported levels of work dissatisfaction (87).

Moreover, the findings suggest that nurses employed in Al-Salt had considerably elevated QWL ratings in comparison to their counterparts working in Amman. This may be attributed to several causes, including disparities in workload, work environment, and work culture. It is conceivable that nurses employed at Al-Salt would experience an improved work-life equilibrium because of a lower patient load or a more nurturing work atmosphere. On the other hand, nurses employed in Amman may encounter heightened levels of stress and workload as a result of the larger number of patients and the presence of more intricate situations. These results emphasize the significance of taking into account the work environment when assessing the quality of work life for nurses. The results of this research indicate that the desire to quit among primary care nurses and midwives in Jordan is a significant factor that might have a detrimental effect on their QWL. This aligns with other studies that have shown an inverse correlation between the desire to quit and work satisfaction (88, 89). Nurses contemplating departure from their present occupation, or the nursing field may encounter diminished levels of job satisfaction, engagement, and overall QWL.

Nonetheless, the factors contributing to the desire to quit among Jordanian primary care nurses and midwives may differ and need investigation in future studies. Work overload, insufficient salary, limited career advancement prospects, unfavorable working circumstances, and incidents of workplace violence are potential factors that may lead to nurses’ intent to leave their jobs (90, 106). Furthermore, personal attributes such as personality characteristics and work ideals may also have an influence (91).

Future studies should focus on identifying the fundamental causes that lead to the intention to quit among primary care nurses and midwives in Jordan. This will enable the development of effective methods to address these problems and enhance the quality of work life. Potential avenues for study might include administering qualitative interviews or organizing focus groups with nurses to get a more comprehensive comprehension of their experiences, attitudes, and perspectives. Longitudinal studies may also be conducted to examine the impact of variables like burnout, stress, and work satisfaction on nurse turnover intentions over some time.

## 5 Implications and recommendations

The findings of this study have significant implications for practitioners in the nursing profession, including nurses, nurse managers, policymakers, and patients. Firstly, the study revealed that primary care nurses and midwives perceive a moderate level of PE and QWL. Therefore, it is recommended that nursing administrators and policymakers take the necessary steps to improve the work environment and conditions of primary care nurses and midwives in Jordan. One possible strategy is to offer training programs aimed at enhancing the sense of empowerment and motivation among nurses. This can be achieved by providing opportunities for nurses to develop new skills, gain new knowledge, and take on leadership roles in their workplaces. Additionally, nurse managers should make efforts to ensure that nurses have access to the necessary resources and support to perform their duties effectively and efficiently (104).

Secondly, the study revealed that the QWL of primary care nurses and midwives in Jordan varies based on their educational level and place of employment. This implies that nursing managers and policymakers should consider developing and implementing targeted interventions to improve the work conditions and environments of nurses working in various locations and educational backgrounds. For instance, nursing administrators can offer incentives or create opportunities for nurses to advance their education, which can lead to a more favorable work environment and better patient outcomes. Furthermore, policymakers should collaborate with healthcare providers and relevant stakeholders to establish policies and procedures that promote work-life balance, flexible working schedules, and support for nurses who are experiencing burnout or stress. Nursing organizations have the opportunity to speak for their members as a whole on matters of utmost importance, such as nurse quality of work life and psychological empowerment, by identifying the difficulties and promoting solutions. By providing an evidence-based range of solutions that can be tailored to the requirements of members within their organizations, they play a crucial role in reshaping the narrative about the difficulties nurses confront to avoid perpetuating a victim attitude.

Thirdly, the finding that intention to leave is negatively associated with QWL has implications for nursing managers, policymakers, and patients. It is essential to develop strategies that can reduce turnover rates among primary care nurses and midwives in Jordan. One possible solution is to offer financial incentives, such as bonuses or salary increases, for nurses who have a high level of job satisfaction and are committed to their work. Additionally, nursing administrators should prioritize creating a supportive and inclusive work culture, where nurses feel valued and respected, and where they have opportunities for career growth and development.

Finally, patients also benefit from the findings of this study. The QWL of nurses is positively correlated with patient outcomes, such as patient satisfaction and quality of care. Therefore, healthcare providers and policymakers need to invest in initiatives that prioritize improving the working conditions and environments of nurses in Jordan. This will not only benefit nurses but also enhance the quality of care provided to patients.

The findings of this study have several implications for scholars and future research. This study highlights the need for further investigation into the factors that contribute to the psychological empowerment and quality of nurse work life of Jordanian primary care nurses and midwives. Future studies could explore the role of specific organizational and environmental factors that may affect these outcomes, such as leadership style, communication patterns, workload, and the physical work environment. In addition, this study contributes to the limited body of literature on the psychological empowerment and quality of work life among nurses and healthcare professionals in the Middle East. Given the unique cultural and societal contexts in this region, there is a need for further research that explores the specific challenges and opportunities faced by healthcare professionals in this context.

The significant differences in QWL scores based on educational level and place of employment suggest that these factors may be important in shaping the work experiences of nurses and midwives in Jordan. Further research could investigate the mechanisms through which education and work location influence QWL and explore potential interventions to improve the work experiences of nurses and midwives in these contexts.

Based on the study findings, several practical recommendations can be made for improving the PE and QWL. Nurse managers should prioritize creating a work environment that promotes PE by providing opportunities for professional development and growth, encouraging autonomy and decision-making, and recognizing and rewarding nurses for their accomplishments. Additionally, managers should consider reducing the workload and providing support and resources to nurses, particularly those who work long hours or have higher workloads. Policy makers should recognize the importance of investing in nursing education and training programs that emphasize the development of PE skills and competencies. This can include courses on leadership, decision-making, and critical thinking, as well as programs that promote collaboration and teamwork among nurses. Efforts should be made to improve the QWL of primary care nurses and midwives by addressing factors such as pay, benefits, workload, and working conditions. Policymakers should work with healthcare organizations to establish policies and guidelines that ensure fair compensation, reasonable work hours, and safe working conditions.

One of the main strengths of this study is its focus on the PE and quality of nurse work life of primary care nurses and midwives in Jordan. This is a relatively understudied population in the literature, and the findings provide valuable insight into the experiences of this group of healthcare professionals. Another strength of the study is its use of validated scales to measure psychological empowerment and quality of nurse work life, which enhances the validity and reliability of the results. The sample size of 273 participants is also relatively large, which increases the generalizability of the findings to similar populations in Jordan and potentially other countries in the Middle East region.

Several limitations should be considered when interpreting the results of this study. The research was confined to primary care nurses and midwives in Jordan, potentially limiting the generalizability of the findings to other healthcare settings or countries. The use of self-reported measures could introduce social desirability bias, as participants might have provided socially acceptable responses. Additionally, the study did not explore the specific reasons underlying the differences in QWL based on participants’ educational levels and workplace settings, leaving room for future research to explore this aspect further. Finally, the study did not investigate other potentially influential variables, such as workload, job demands, and social support, which could impact both psychological empowerment and the quality of nurses’ work lives. So, future research must specify the relationship between PE and QWL using demographic sociological variables with statistical differences as control variables using conceptual or theoretical frameworks. One additional limitation to consider is that the survey instrument, BQNWL, was originally developed for nurses and may not fully capture the unique experiences and perspectives of midwives, potentially affecting the applicability of the results to this specific professional group.

## 6 Conclusion

In conclusion, this study aimed to assess the relationship between PE and QWL among Jordanian primary care nurses and midwives. The study utilized a cross-sectional design with a sample of 273 nurses. The results showed that the levels of PE and QWL among Jordanian primary care nurses and midwives need improvement, particularly in the areas of meaning and self-determination in the PE scale, and the Work World Dimension subscale in the QWL scale. The low scores on the meaning and self-determination PE subscales indicated that the nurses feel a lack of purpose and autonomy in their work, consistent with previous research on nursing job satisfaction internationally and in the region. The nursing profession is known to be a highly demanding and stressful job, which can lead to feelings of burnout and a lack of meaning in work. Therefore, the current study findings highlight the need for interventions supporting nurses’ sense of meaning and autonomy at work to improve their PE.

Additionally, the study found a significant positive relationship between PE and QWL. Besides, there were significant differences in QWL scores based on educational level and place of employment and a significant negative relationship between intention to leave and QWL.

The study’s findings have several implications for nursing practitioners, nurse managers, policymakers, patients, and scholars. Practical recommendations include the need to prioritize interventions to improve the QWL and PE levels of primary care nurses and midwives in Jordan, through providing opportunities for professional development, job autonomy, and increasing nurses’ involvement in decision-making processes.

## Data Availability

The original contributions presented in the study are included in the article/supplementary material, further inquiries can be directed to the corresponding author.
